# Supplementary material for: Adaptive stretching of representations across brain regions and deep learning model layers
Source: Nat Commun. 2025 Nov 21;16:10302. doi: 10.1038/s41467-025-65231-y (PMC12638828; doi:10.1038/s41467-025-65231-y)
Supplement: Supplementary file 1 — Supplementary Information [file 41467_2025_65231_MOESM1_ESM.pdf]

Supplementary Information for

Adaptive stretching of representations across brain regions and deep learning model layers

Xin-Ya Zhang, Sebastian Bobadilla-Suarez, Xiaoliang Luo,
Marilena Lemonari, Scott L. Brincat, Markus Siegel,
Earl K. Miller, Bradley C. Love

This PDF file includes:
Supplementary Methods
Supplementary Figures S1 to S18
Supplementary Tables S1 to S17
References

Contents

I Experiment setting **2**
I.1 Simulating trials 2
I.2 Stimulus image details 2

II Neural coding measures **3**
II.1 Rate coding 3
II.2 Temporal coding 3

III Supplementary results **6**
III.1 ANN model architecture and performance 6
III.2 Identify the best corresponding neural measure 8
III.3 Dimensional stretching on 24 pairs (ANOVA towards regions measured by ISI-distance) 13
III.4 Dimensional stretching on 24 pairs (ANOVA towards regions measured by rate coding) 15
III.5 Dimensional stretching on 24 pairs (ANOVA towards LSTM layers) 18
III.6 Cognitive model-based analyses (ANOVA towards brain regions) 20
III.7 Cognitive model-based analyses (ANOVA towards LSTM layers) 21
III.8 ANOVA analysis to compare the brain and the CNN-LSTM model 24
III.9 Additional results 24

I Experiment setting

I.1 Simulating trials

In the original experiment [1], two rhesus monkeys, one female (Paula) and one male (Rex), were trained to perform two categorization tasks. Monkeys learned to categorize stimuli into different classes indicated by the task cue. The cued task was color in half the trials and motion in the other half. Specifically, monkeys were shown a sequence of animated movies that are made of a fixation point (0.5 s), a cue (1 s), and stimulus (up to 3 s) in each trial. The cue reflects whether stimuli should be categorized by its motion or color. There are four types of cues, namely the cross, the quatrefoil, the circle and the triangle cue. The cross and the quatrefoil indicate that stimuli categorization should be made based on motion and the circle and the triangle indicate stimuli categorization should be made based on color. After cueing, the cue disappeared and the stimulus will be presented at the center of the fixation spot. Monkeys were free to respond any time up to 3 s after stimulus onset. During learning, neuronal activity was recorded in six regions of the brain across a maximum of 108 electrodes simultaneously. Images of the fixation point and cue are static in movies, while stimulus images are moving, made of 400 moving dots with one out of 21 color-motion combinations and one out of 2 dot's speed. There are 21 color-motion stimuli from 7 possible colors and 7 possible motion directions (Fig. 1c). For correct responses to categorization, monkeys were rewarded with apple juice. There are 16 color-motion stimuli not near the category boundary but 5 ambiguous stimuli items on or near the category boundary. Monkeys were always rewarded for not giving any responses in ambiguous trials (stimuli on the category boundary). These ambiguous trials were excluded for calculating the animals' percent correct performance in the original experiment.

To train our deep learning model, we construct identical fixation and cue images to the ones in the original experiment and stimulus images with the same stimuli patterns (stimulus diameter, dot diameter, number of dots and dot speed). We have a total of 6720 trials covering 4 cues, 2 speeds, 21 color-motion stimuli with 40 random stimulus dots' initial positions ($4 \times 2 \times 21 \times 40 = 6720$). Since images of the fixation point and cue are static, we repeat the same image to simulate the image sequences shown to monkeys, while the stimuli dots are moving, thus we choose a frequency of 60 frames per second to make it look continuous, that is a total of 270 images with 30 images of the fixation point, 60 images of cue and 180 images of stimuli in each trial.

I.2 Stimulus image details

We construct 128×128 pixels RGB images. In the fixation point image, the distance between the fixation point and either of the grey dots is equal to 1/4 of the horizontal length of the image. This ratio is kept constant for the rest of the image types. We import the monochrome cue images and changed the cue color to grey, the background color to black, adding the three dots also appearing in the fixation point image. Stimuli are colored dynamic random dot patterns with 100% motion coherence presented centrally on the fixation spot (stimulus diameter: 3.2; dot diameter: 0.08; number of dots: 400; dot speed: 1.67 /s or 10 /s for half of the recording trials, respectively). All colors are defined in the CIE L*a*b* space and had the same luminance and saturation.

II Neural coding measures

In this work, we consider candidate neural dissimilarity measures, such as rate coding, temporal coding (e.g., ISI distance and SPIKE distance). Rate coding utilizes a simple scheme where total spike counts alone carry information. Temporal coding measures such as ISI distance and SPIKE distance focus on the temporal profiles of brain activity. ISI distance measures relative spike timing to see whether rhythms and patterns match, while SPIKE distance also incorporates the absolute timing of spikes which can be useful for evaluating synchrony between spike trains.

II.1 Rate coding

Rate coding represents stimulus as neural firing rate across neurons in brain regions or lobes over a period of time. It is assumed that a time bin with higher firing rate represents more information. To construct rate-coding-based RDM, we first represent each stimulus as a n_r-dimensional vector, where n_r is the number of recording sites. Each element of the vector corresponds to the firing rate of a specific neuron/electrode in a time bin. To maintain uniform dimensionality for calculating dissimilarity between stimulus vectors, we used 30 recording sites per brain region, resulting in a total of $n_r = 180$ recording sites (sites with no recordings during a trial were excluded from the analysis). We then compute pairwisedistance/dissimilarity between 16 stimuli using a range of distance/dissimilarity measures including Euclidean, Cosine distance and Pearson correlation which fill the entries of a RDM.

II.2 Temporal coding

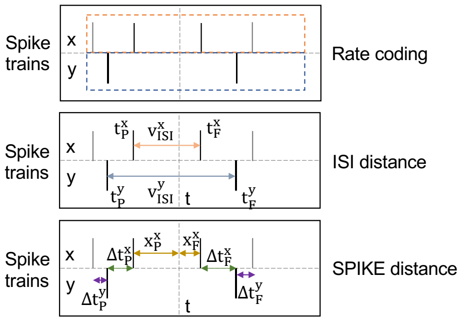

Figure S1: The illustration of spike trains dissimilarity measures. Rate coding (the first row) represents a stimulus as the total number of spikes across neurons. ISI distance (the second row) compares two stimuli based on the relative spike interval $\mathrm{v}^x_{\mathrm{ISI}}$ and $\mathrm{v}^y_{\mathrm{ISI}}$ while SPIKE distance (the third row) compares two stimuli, not only based on the inter-spike interval but also on the absolute spike timing (e.g., $\Delta \mathrm{t_P}, \Delta \mathrm{t_F}, \mathrm{x_P}$ and $\mathrm{x_F}$).

ISI distance and SPIKE distance directly calculate the distances between stimuli pairs from spike trains. With differences which we will note below, both measures can be defined as the

temporal average of the respective time profile

$$\mathrm{D_X} = \int_{t=0}^{T} |\mathrm{X}(t)|dt, \quad \mathrm{X} = I, K \tag{S1}$$

where I denotes ISI distance function and K denotes SPIKE distance function.

For each spike train $u \in \{x, y\}$, at each time instant (Supplementary Figure S1), the time of the previous spike is

$$\mathrm{t_P^{\mathit{u}}}(t) = \max\left(t_i^u |\, t_i^u \leq t\right)$$

and the time of the following spike is

$$\mathrm{t_F^{\mathit{u}}}(t) = \min\left(t_i^u |\, t_i^u > t\right)$$

as well as the interspike interval is

$$\mathrm{v_{ISI}^{\mathit{u}}}(t) = \mathrm{t_F^{\mathit{u}}}(t) - \mathrm{t_P^{\mathit{u}}}(t)$$

ISI distance focuses on the relative firing rate pattern based on the instantaneous interspike intervals [2]. The ISI distance between spike trains x and y at time instant t is calculated as

$$I(t) = \begin{cases} \frac{\mathrm{v_{ISI}^{\mathit{x}}}(t)}{\mathrm{v_{ISI}^{\mathit{y}}}(t)} - 1 & \text{if } \mathrm{v_{ISI}^{\mathit{x}}}(t) \leq \mathrm{v_{ISI}^{\mathit{y}}}(t) \\ -\left(\frac{\mathrm{v_{ISI}^{y}}(t)}{\mathrm{v_{ISI}^{x}}(t)} - 1\right) & \text{else} \end{cases}.$$

For spike trains which are identical or have constant and equal interspike intervals with phase shift, the ISI distance reaches 0. ISI distance approaches -1 or 1 if the first or the second spike train is much faster than the other. We derive ISI-distance-based RDM consisting of ISI distance between 16 stimuli by temporal averaging over the absolute values $|I(t)|$ in the time bin using Eq. S1.

SPIKE distance builds upon the properties of the ISI distance with a specific focus on absolute spike timing [3], which accounts for both firing rate differences as well as precise spike time differences.

The SPIKE distance profile is calculated in two steps: first, a spike time difference is calculated for each spike and then for each time instant the relevant spike time differences are selected, weighted, and normalized. Specifically, for two spike trains x and y, each time instant is surrounded by four spikes: the preceding spike from x spike train $\mathrm{t_P^{\mathit{x}}}$, the following spike from x spike train $\mathrm{t_F^{\mathit{x}}}$, the preceding spike from y spike train $\mathrm{t_P^{\mathit{y}}}$ and the following spike from y spike train $\mathrm{t_F^{\mathit{y}}}$. Each of these spikes can be identified with a spike time difference to the nearest spike in the other spike train, for example, the instantaneous differences of previous and following spike times are denoted as

$$\Delta\mathrm{t_P^{\mathit{x}}}(t) = \min_i(|\mathrm{t_P^{\mathit{x}}}(t) - \mathrm{t}_i^{\mathit{y}}|),$$

and analogously for $\Delta\mathrm{t_F^{\mathit{x}}}$, $\Delta\mathrm{t_P^{\mathit{y}}}$ and $\Delta\mathrm{t_F^{\mathit{y}}}$ (Supplementary Figure S1). For spike train $u \in \{x, y\}$, the intervals from the time instant under consideration to the previous and the following spikes is

$$\mathrm{x_P^{\mathit{u}}}(t) = t - \mathrm{t_P^{\mathit{u}}}(t) \quad \text{and} \quad \mathrm{x_F^{\mathit{u}}}(t) = \mathrm{t_F^{\mathit{u}}}(t) - t.$$

The local weighting for the spike time differences of the spike train $u \in \{x, y\}$ is

$$K_u(t) = \frac{\Delta \mathrm{t}_{\mathrm{P}}^{(u)}(t)\mathrm{x}_{\mathrm{F}}^{(u)}(t) + \Delta \mathrm{t}_{\mathrm{F}}^{(u)}(t)\mathrm{x}_{\mathrm{P}}^{(u)}(t)}{\mathrm{v}_{\mathrm{ISI}}^{u}(t)}$$

which weights the spike time differences for each spike train according to the relative distance of the corner spike from the time instant under consideration. In the last step, the two contributions of the two spike trains are locally weighted by their instantaneous interspike intervals, and then the SPIKE distance between the spike trains x and y at time instant t is calculated as

$$K(t) = \frac{K_x(t)\mathrm{v}_{\mathrm{ISI}}^{y}(t) + K_y(t)\mathrm{v}_{\mathrm{ISI}}^{x}(t)}{2\left\langle \mathrm{v}_{\mathrm{ISI}}^{(u)}(t) \right\rangle_u^2}$$

which ranges from 0 to 1. A value of 0 indicates identical spike trains and 1 indicates maximal dissimilarity. Similarly, we derive SPIKE-distance-based RDM consisting of SPIKE distance between 16 stimuli by temporal averaging over the absolute values $|K(t)|$ in the time bin using Eq. S1.

Both ISI and SPIKE distances are calculated for each recording site and then averaged across all sites. Unlike rate coding, ISI and SPIKE distances are not affected by empty recording sites. Since empty sites return NaN values when no data is recorded, they are excluded from the calculations.

III Supplementary results

III.1 ANN model architecture and performance

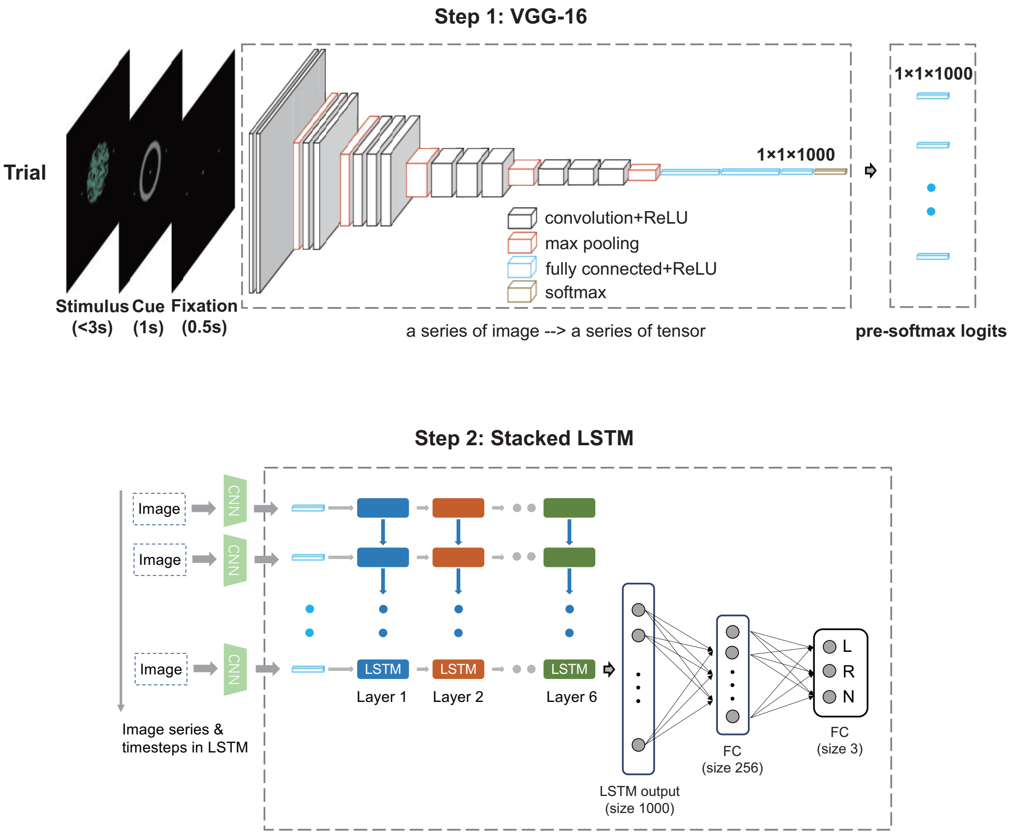

Figure S2: **The architecture of the CNN-LSTM model.** In our artificial neural network (ANN), a pre-trained VGG-16 maps each two-dimensional image to a pre-softmax tensor of size 1000. The output of the CNN, which is the pre-softmax logits, serves as input to a six-layer stacked LSTM. To transform the LSTM output into the decision response, we apply two fully connected (FC) linear transformations: the first with 256 units and the second with 3 units (L, R and no response).

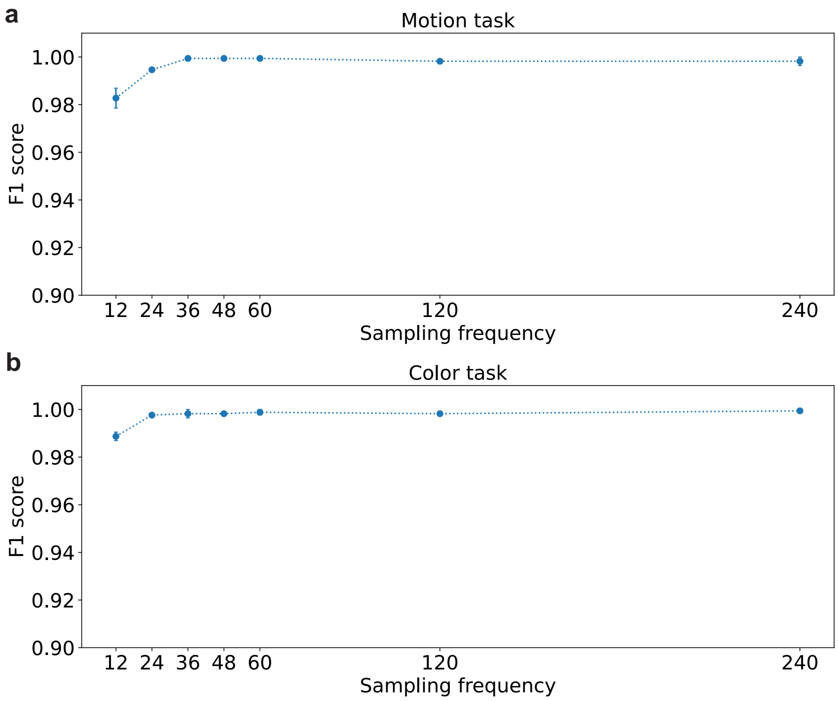

Figure S3: **Model performance in different sampling frequency.** Panels **a** and **b** show the F1 score for the motion task (**a**) and the color task (**b**), respectively. Each dot represents one chosen sampling frequency (i.e., frames per second) for stimulus images, and the performance score is averaged over task-relevant trails at default learning rate. The original experiment reported accuracy of 94% and 89% for the motion and color tasks in the monkey brain, respectively. In contrast, our CNN-LSTM model achieves a higher accuracy of 98% at least.

III.2 Identify the best corresponding neural measure

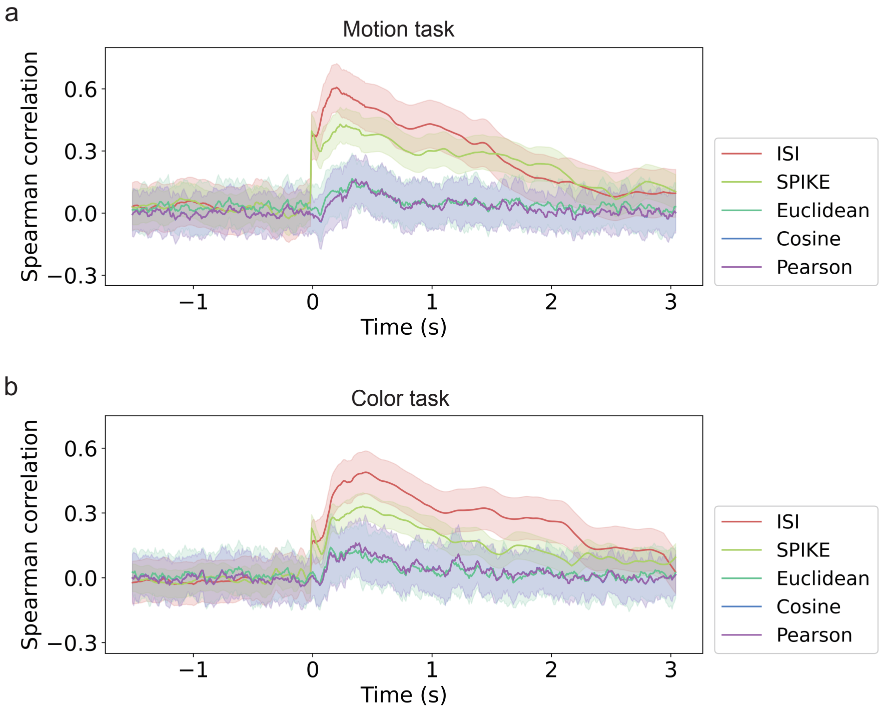

Figure S4: **Spearman correlation for each spiking measures in the whole trial with a 50 ms sliding window**. Panels **a** and **b** correspond to the motion and color tasks, respectively. Time bins are 50 ms wide, moving with a 10 ms step, and start from the indicated time. Higher correlation indicates better agreement. Spiking timing measures best capture the experimenter intended coordinates. The band around each dissimilarity measure depicts the standard variance across sessions (95% confidence interval is used in the main text; one session includes multiple trials with same position of electrodes, see Methods for details). Time on the horizontal axis is the lower bound of the time window.

Table S1: Two-tailed t-test with Bonferroni correction $\alpha = 0.05/n = 0.0125$ ($n = 4$) was conducted to show that the ISI measure performed best. The analysis was conducted on 250 ms period data with a 50 ms sliding window, starting at 0 s. Notice we averaged the correlation between motion and color tasks to evaluate overall performance, and this approach was applied in subsequent pairwise t-test comparisons.

250 ms period data (50 ms sliding window)	ISI
SPIKE	t(21) $= 4.80$; $p = 6.7e^{-5}$
Euclidean	t(21) $= 14.58$; $p = 3.8e^{-18}$
Cosine	t(21) $= 15.95$; $p = 1.7e^{-18}$
Pearson	t(21) $= 15.99$; $p = 1.3e^{-18}$

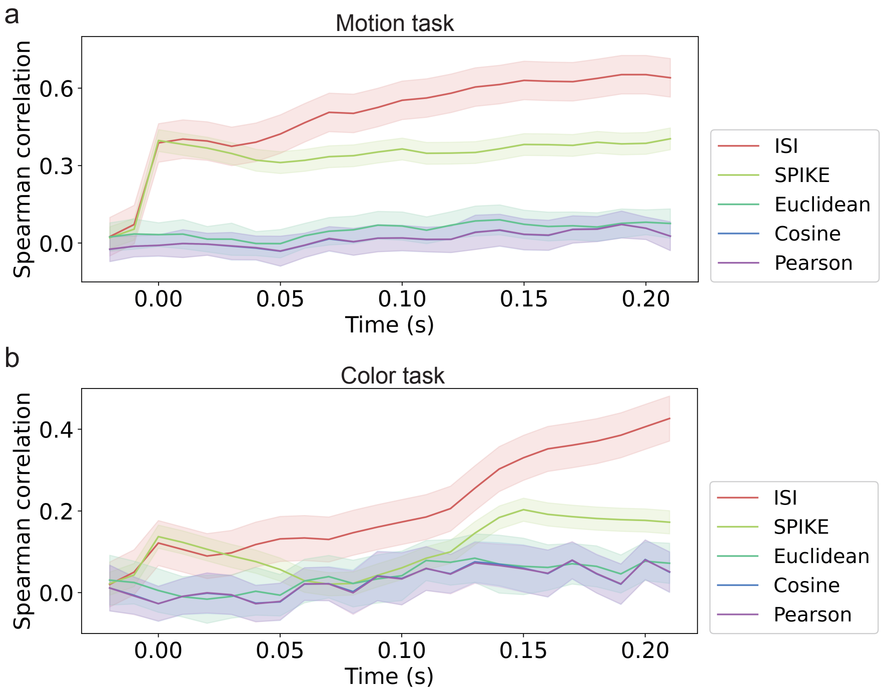

Figure S5: **Spike timing measures best capture the experimenter intended coordinates when using a 20 ms sliding window.** Top panel (**a**) shows the motion task, whereas the bottom panel (**b**) shows the color task. We found ISI measure surpasses other measures in a 20 ms sliding window (A one-way ANOVA analysis is conducted to show the significance of different types of measures from stimulus onset, $F(4, 105) = 180.79, p < .0001$. Two-tailed t-test with Bonferroni correction was shown in Table S2). A 50 ms time window is shown in the main text, indicating the length of time window chosen does not affect the advantage of timing measures in assessing spike trains in such categorization tasks.

Table S2: Two-tailed t-test with Bonferroni correction $\alpha = 0.05/n = 0.0125$ $(n = 4)$ was conducted to show that the ISI measure performed best (250 ms period data with a 20 ms sliding window, beginning at 0 s) in pairwise comparisons.

250 ms period data (20 ms sliding window)	ISI
SPIKE	t(21) $= 5.80$; $p = 7.6e^{-7}$
Euclidean	t(21) $= 14.22$; $p = 2.6e^{-16}$
Cosine	t(21) $= 15.14$; $p = 3.5e^{-18}$
Pearson	t(21) $= 15.15$; $p = 1.4e^{-17}$

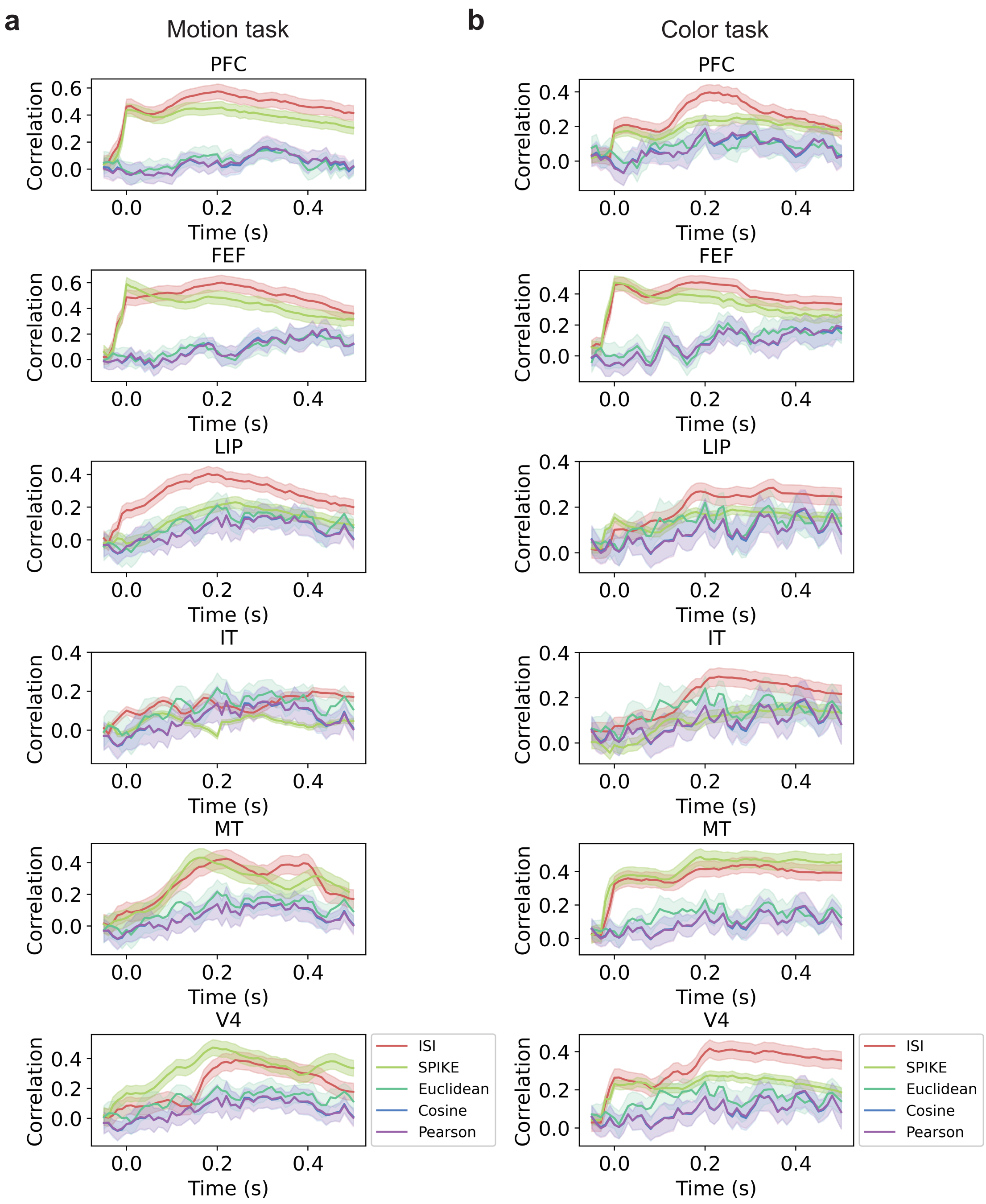

Figure S6: **Spike timing matters in each individual brain region with a 50 ms sliding window.** Results are shown separately for the motion task (**a**) and the color task (**b**). Spike timing measures best capture the experimenter-designed coordinates in six brain regions (two-tailed t-test with Bonferroni correction; see Tables S3–8).

Table S3: The type of measure matters in region PFC ($F(4, 105) = 166.85, p < .0001$). Two-tailed t-test with Bonferroni correction $\alpha = 0.05/n = 0.0125$ ($n = 4$) was conducted to show that the ISI measure performed best in pairwise comparisons in the PFC using 250 ms period data with a 50 ms sliding window, beginning at 0 s.

250 ms period data in PFC (50 ms sliding window)	ISI
SPIKE	t(21) = 4.25; p = .00012
Euclidean(roi)	t(21) = 19.71; p=7.6e^{-23}
Cosine(roi)	t(21) = 13.42; p=8.9e^{-17}
Pearson(roi)	t(21) = 13.89; p=2.7e^{-17}

Table S4: The type of measure matters in region FEF ($F(4, 105) = 303.64.1, p < .0001$). Two-tailed t-test with Bonferroni correction $\alpha = 0.05/n = 0.0125$ ($n = 4$) was conducted to show that the ISI measure performed best in pairwise comparisons in the FEF using 250 ms period data with a 50 ms sliding window, beginning at 0 s.

250 ms period data in FEF (50 ms sliding window)	ISI
SPIKE	t(21) = 3.53; p = .0010
Euclidean(roi)	t(21) = 23.77; p=5.6e^{-26}
Cosine(roi)	t(21) = 25.25; p=5.1e^{-27}
Pearson(roi)	t(21) = 25.64; p=2.8e^{-27}

Table S5: The type of measure matters in region LIP ($F(4, 105) = 51.05, p < .0001$). Two-tailed t-test with Bonferroni correction $\alpha = 0.05/n = 0.0125$ ($n = 4$) was conducted to show that the ISI measure performed best in pairwise comparisons in the LIP using 250 ms period data with a 50 ms sliding window, beginning at 0 s.

250 ms period data in LIP (50 ms sliding window)	ISI
SPIKE	t(21) = 6.45; p=8.9e^{-8}
Euclidean(roi)	t(21) = 10.16; p=6.9e^{-13}
Cosine(roi)	t(21) = 10.94; p=7.2e^{-14}
Pearson(roi)	t(21) = 11.04; p=5.4e^{-14}

Table S6: The type of measure matters in region IT ($F(4, 105) = 15.87, p < .0001$). Two-tailed t-test with Bonferroni correction $\alpha = 0.05/n = 0.0125$ ($n = 4$) was conducted to show that the ISI measure performed best in pairwise comparisons in the IT using 250 ms period data with a 50 ms sliding window, beginning at 0 s.

250 ms period data in IT (50 ms sliding window)	ISI
SPIKE	t(21) = 7.67; p=1.6e^{-9}
Euclidean(roi)	t(21) = 4.78; p=2.1e^{-5}
Cosine(roi)	t(21) = 7.19; p=7.8e^{-9}
Pearson(roi)	t(21) = 7.30; p=5.5e^{-9}

Table S7: The type of measure matters in region MT ($F(4, 105) = 100.17, p < .0001$). Two-tailed t-test with Bonferroni correction $\alpha = 0.05/n = 0.0125$ ($n = 4$) was conducted to show that the ISI measure performed best in pairwise comparisons in the MT using 250 ms period data with a 50 ms sliding window, beginning at 0 s.

250 ms period data in MT (50 ms sliding window)	ISI
SPIKE	t(21) = -0.54; p=.5947
Euclidean(roi)	t(21) = 12.42; p=1.2e^{-15}
Cosine(roi)	t(21) = 13.95; p=2.3e^{-17}
Pearson(roi)	t(21) = 14.06; p=1.8e^{-17}

Table S8: The type of measure matters in region V4 ($F(4, 105) = 67.22, p < .0001$). Two-tailed t-test with Bonferroni correction $\alpha = 0.05/n = 0.0125$ ($n = 4$) was conducted to show that the ISI measure performed best in pairwise comparisons in the V4 using 250 ms period data with a 50 ms wide sliding window, beginning at 0 s.

250 ms period data in V4 (50 ms sliding window)	ISI
SPIKE	t(21) = -2.54; p=.0139
Euclidean(roi)	t(21) = 7.13; p=9.5e^{-9}
Cosine(roi)	t(21) = 9.48; p=5.4e^{-12}
Pearson(roi)	t(21) = 9.56; p=4.2e^{-12}

III.3 Dimensional stretching on 24 pairs (ANOVA towards regions measured by ISI-distance)

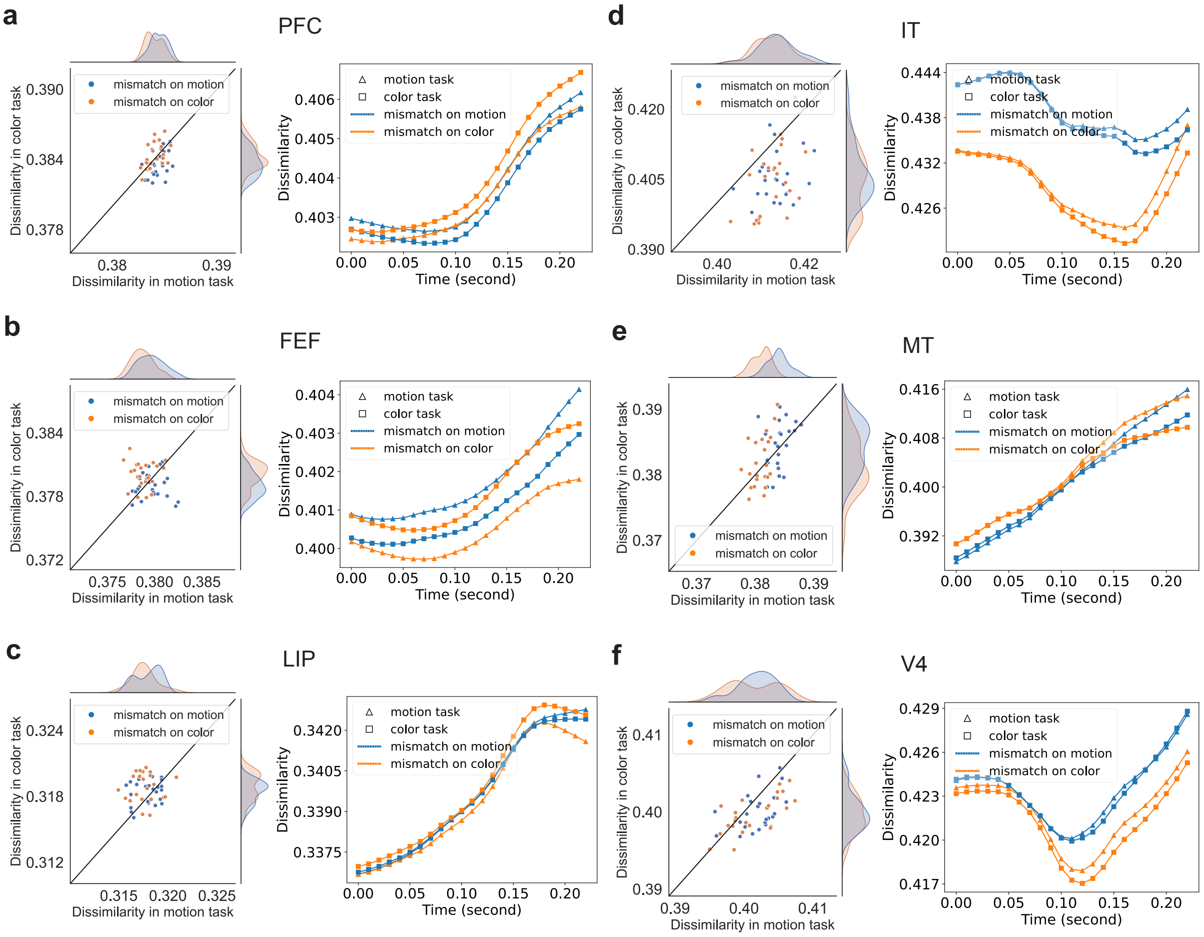

Figure S7: **Mismatching pairs in neural data measured by ISI-distance across brain areas (50 ms sliding window).** Panels **a–f** correspond to brain regions PFC (**a**), FEF (**b**), LIP (**c**), IT (**d**), MT (**e**), and V4 (**f**), respectively. Regions PFC, FEF and LIP show clear qualitative pattern of performance (i.e., stimulus pairs that mismatch on the task relevant dimension) while regions IT and V4 are inclined to task context. A two-way repeated ANOVA was conducted with fixed factors (mismatching and task) and a random effect (subjects), indicating interaction item is significant in regions PFC, FEF and LIP (for 250 ms period data see Table S9, for extended 500 ms period data see Table S10).

Table S9: A two-way repeated measure ANOVA with fixed factors (motion/color mismatch and motion/color task) and a random effect (subjects) was conducted on 24 stimulus pairs. The dissimilarity between stimuli in 250 ms period data was measured by ISI-distance. Task context significantly affects the dissimilarity in each region, with the interaction term being significant in the whole brain, as well as in regions PFC, FEF and LIP.

Two-way ANOVA (250 ms period data)	PFC	FEF	LIP	IT	MT	V4	All
motion/color mismatch	$F_{1,4411}$=2.76 p=0.0967	$F_{1,4411}$=5.83 p=0.0158 (*)	$F_{1,4411}$=0.58 p=0.4470 (*)	$F_{1,4411}$=9.96 p=0.0016 (**)	$F_{1,4411}$=36.76 p=1.4e^{-9} (****)	$F_{1,4411}$=0.936 p=0.3330	$F_{1,4411}$=0.038 p=0.845
motion/color task	$F_{1,4411}$=67.41 p=2.5e^{-16} (****)	$F_{1,4411}$=374.7 p< 2e^{-16} (****)	$F_{1,4411}$=250.4 p< 2e^{-16} (****)	$F_{1,4411}$=913.7 p< 2e^{-16} (****)	$F_{1,4411}$=142.2 p< 2e^{-16} (****)	$F_{1,4411}$=1021 p< 2e^{-16} (****)	$F_{1,4411}$=33.56 p=7.2e^{-9} (****)
interaction	$F_{1,4411}$=131.3 p< 2e^{-16} (****)	$F_{1,4411}$=389.3 p< 2e^{-16} (****)	$F_{1,4411}$=25.58 p=4.3e^{-7} (****)	$F_{1,4411}$=2.536 p=0.1113	$F_{1,4411}$=0.27 p=0.6030	$F_{1,4411}$=0.113 p=0.7360	$F_{1,4411}$=132.0 p< 2e^{-16} (****)

Table S10: A two-way repeated measure ANOVA result for 500 ms period data. The findings are consistent with the 250 ms period (Table S9), showing stretching effect of task-relevant context and interaction in the whole brain, PFC, FEF and LIP.

Two-way ANOVA (500 ms period data)	PFC	FEF	LIP	IT	MT	V4	All
motion/color mismatch	$F_{1,8827}$=8.222 p=0.0042 (**)	$F_{1,8827}$=349.2 p< 2e^{-16} (****)	$F_{1,8827}$=49.67 p=2.0e^{-12} (****)	$F_{1,8827}$=3.272 p=0.0705	$F_{1,8827}$=113.7 p< 2e^{-16} (****)	$F_{1,8827}$=1381 p< 2e^{-16} (****)	$F_{1,8827}$=79.59 p< 2e^{-16} (****)
motion/color task	$F_{1,8827}$=0.731 p=0.3926	$F_{1,8827}$=0.004 p=0.9470	$F_{1,8827}$=19.13 p=1.2e^{-5} (****)	$F_{1,8827}$=184.1 p< 2e^{-16} (****)	$F_{1,8827}$=97.25 p< 2e^{-16} (****)	$F_{1,8827}$=7.433 p=0.0064 (**)	$F_{1,8827}$=0.025 p=0.875
interaction	$F_{1,8827}$=6.803 p=0.0091 (**)	$F_{1,8827}$=16.95 p=3.9e^{-5} (****)	$F_{1,8827}$=140.1 p< 2e^{-16} (****)	$F_{1,8827}$=0.645 p=0.4221	$F_{1,8827}$=0.397 p=0.5290	$F_{1,8827}$=3.624 p=0.0569	$F_{1,8827}$=124.3 p< 2e^{-16} (****)

III.4 Dimensional stretching on 24 pairs (ANOVA towards regions measured by rate coding)

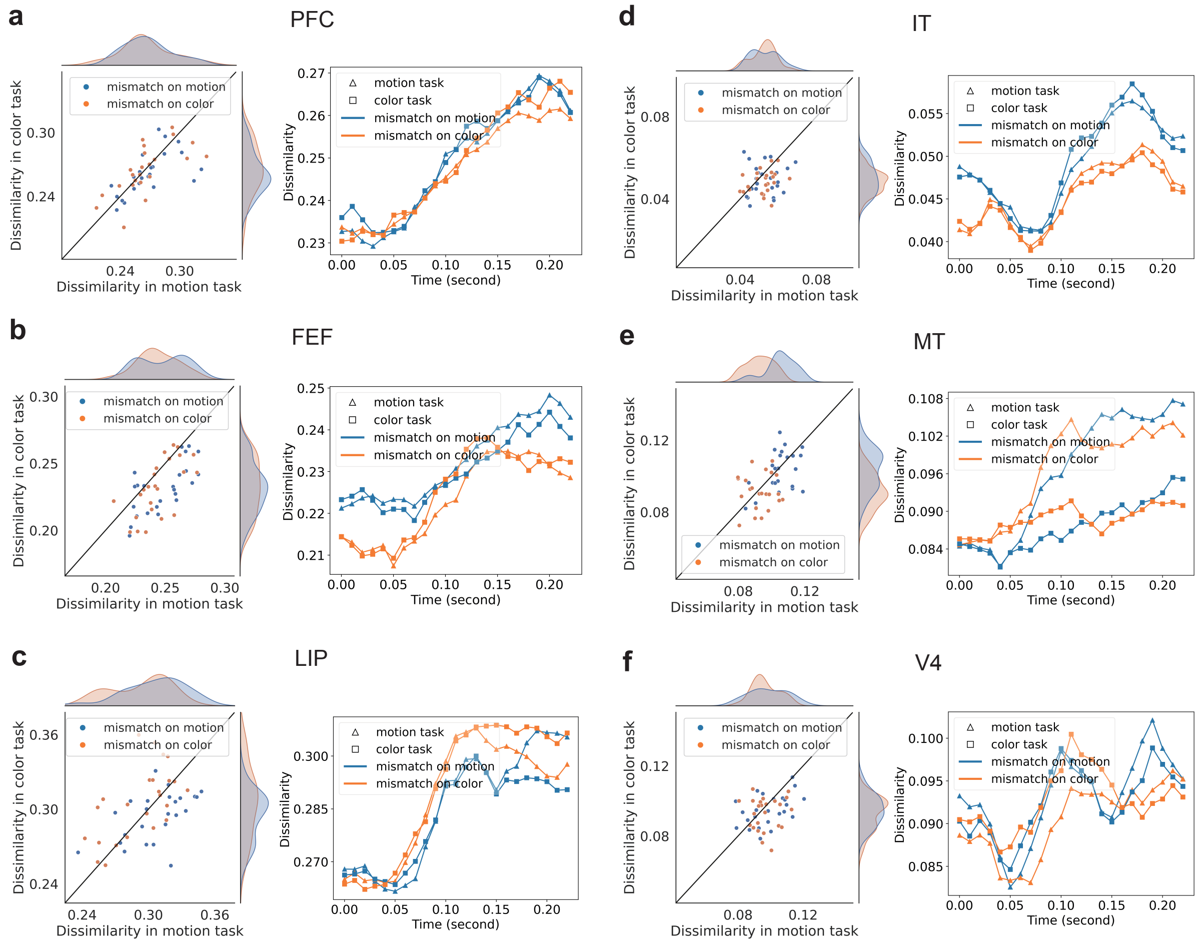

Figure S8: **Mismatching pairs in neural data measured by rate coding (Euclidean distance) across brain areas (50 ms sliding window).** Results are shown for six brain regions: PFC (**a**), FEF (**b**), LIP (**c**), IT (**d**), MT (**e**), and V4 (**f**). A two-way repeated ANOVA was conducted with fixed factors (mismatching and task) and a random effect (subjects), indicating interaction item is significant in regions FEF, LIP and V4 (for 250 ms period data see Table S11, for extended 500 ms period data see Table S12).

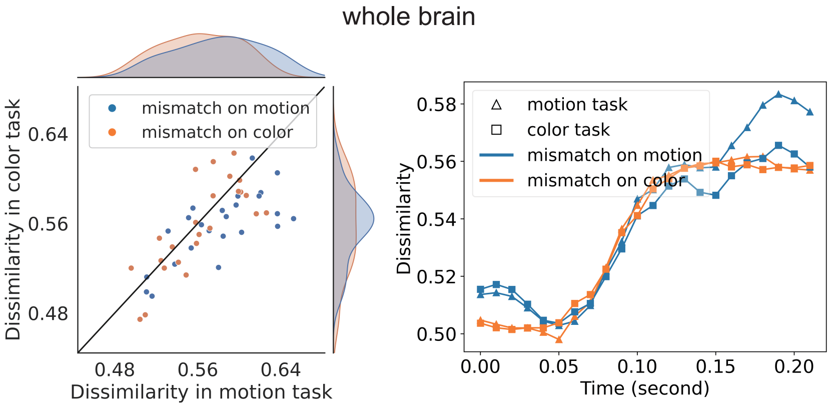

Figure S9: **Mismatching pairs in neural data measured by rate coding (Euclidean distance) in the whole brain (i.e., all six brain regions included).** We used 50 ms sliding window here. The dissimilarity between stimuli in neural data is measured by rate coding (Euclidean distance). A two-way repeated ANOVA was conducted with fixed factors (mismatching and task) and a random effect (subjects), indicating interaction item is significant in the whole brain (for 250 ms period data see Table S11, 500 ms see Table S12).

Table S11: A two-way repeated measure ANOVA with fixed factors (motion/color mismatch and motion/color task) and a random effect (subjects) was conducted on 24 stimulus pairs. The dissimilarity between stimuli in 250 ms period data was measured by rate coding (Euclidean distance). Task context affects the dissimilarity in each region, while the interaction term is significant in the whole brain, as well as regions FEF, LIP, and V4.

Two-way ANOVA (250 ms period data)	PFC	FEF	LIP	IT	MT	V4	All
motion/color mismatch	$F_{1,4411}$=4.179 p=0.0409 (*)	$F_{1,4411}$=0.566 p=0.4517	$F_{1,4411}$=0.005 p=0.9409	$F_{1,4411}$=0.009 p=0.9240	$F_{1,4411}$=131.08 p$< 2e^{-16}$ (****)	$F_{1,4411}$=1.656 p=0.1982	$F_{1,4411}$=9.588 p=0.0019 (**)
motion/color task	$F_{1,4411}$=8.932 p=0.0038 (**)	$F_{1,4411}$=172.0 p$< 2e^{-16}$ (****)	$F_{1,4411}$=59.04 p=$1.6e^{-14}$ (****)	$F_{1,4411}$=67.84 p$< 2e^{-16}$ (****)	$F_{1,4411}$=1.448 p=0.2290	$F_{1,4411}$=3.591 p=0.0581	$F_{1,4411}$=13.15 p=0.0003 (***)
interaction	$F_{1,4411}$=0.155 p=0.6935	$F_{1,4411}$=12.73 p=0.0004 (***)	$F_{1,4411}$=13.07 p=0.0003 (***)	$F_{1,4411}$=0.978 p=0.3230	$F_{1,4411}$=0.430 p=0.5120	$F_{1,4411}$=4.517 p=0.0336 (*)	$F_{1,4411}$=7.947 p=0.0048 (**)

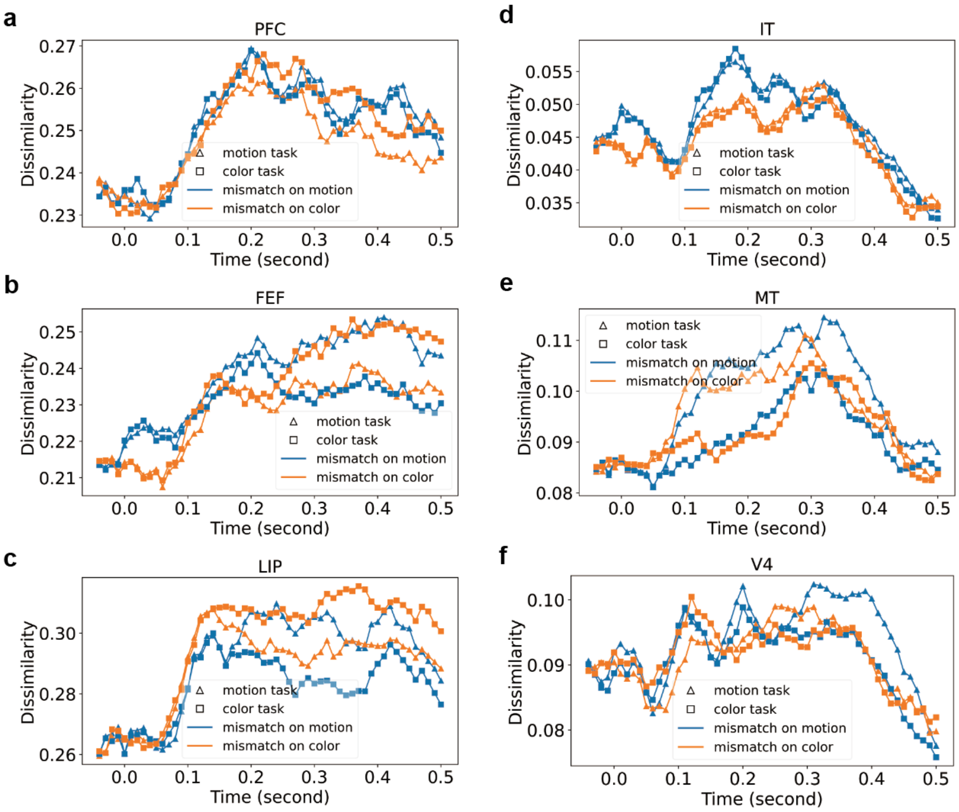

Figure S10: **Extended 500 ms period data results for mismatching pairs measured by rate coding (Euclidean distance) across brain areas (50 ms sliding window).** Left panels (**a**–**c**) show regions PFC, FEF, and LIP, and right panels (**d**–**f**) show IT, MT, and V4. The extended analysis confirms the qualitative patterns observed in 250 ms period data results (Fig. S8), with statistical evaluation based on a two-way repeated ANOVA (see Table S12).

Table S12: A two-way repeated measures ANOVA result for 500 ms period data measured by rate coding, showing the interaction term of the stretching in the whole brain, FEF and LIP.

Two-way ANOVA (500 ms period data)	PFC	FEF	LIP	IT	MT	V4	All
motion/color mismatch	$F_{1,8827}$=0.994 p=0.319	$F_{1,8827}$=2.933 p=0.0868 (*)	$F_{1,8827}$=7.090 p=0.0078 (**)	$F_{1,8827}$=1.948 p=0.163	$F_{1,8827}$=0.046 p=0.8303	$F_{1,8827}$=0.242 p=0.623	$F_{1,8827}$=0.233 p=0.629
motion/color task	$F_{1,8827}$=0.727 p=0.394	$F_{1,8827}$=0.168 p=0.6815	$F_{1,8827}$=0.057 p=0.8120	$F_{1,8827}$=0.130 p=0.718	$F_{1,8827}$=4.401 p=0.0359 (*)	$F_{1,8827}$=0.200 p=0.655	$F_{1,8827}$=1.057 p=0.304
interaction	$F_{1,8827}$=1.081 p=0.298	$F_{1,8827}$=14.72 p=0.0001 (***)	$F_{1,8827}$=14.10 p=0.0002 (***)	$F_{1,8827}$=0.007 p=0.933	$F_{1,8827}$=0.436 p=0.509	$F_{1,8827}$=0.248 p=0.618	$F_{1,8827}$=7.977 p=0.0048 (**)

III.5 Dimensional stretching on 24 pairs (ANOVA towards LSTM layers)

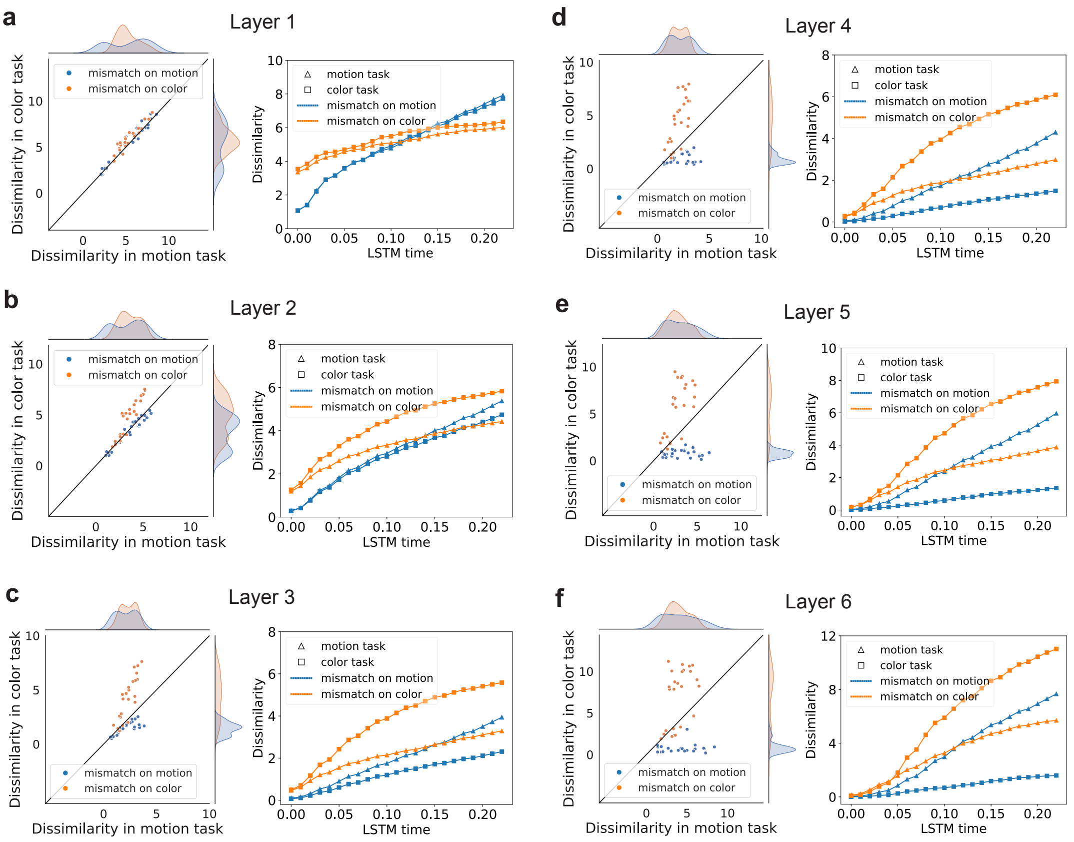

Figure S11: **Mismatching pairs in model representations measured by Euclidean distance.** The dissimilarity between stimuli in model representations is measured by Euclidean distance. Panels **a–f** correspond to layers 1 through 6 of the CNN-LSTM model, respectively. The CNN-LSTM model demonstrates better qualitative pattern of performance (i.e., stimulus pairs that mismatch on the task relevant dimension) at advanced layers. A three-way repeated ANOVA was conducted to analyze the dissimilarity between mismatching dimension, task context and layer number (Table S13).

Table S13: A three-way repeated ANOVA with fixed factors (motion/color mismatch and motion/color task as categorical variables, while layer number as a continuous variable) and a random effect (4 simulations) was conducted to analyze 24 stimulus pairs. The dissimilarity between stimuli in model representations was measured using Euclidean distance.

Three-way ANOVA	motion/color mismatch	motion/color task	layer number	interaction (mismatch: task)	interaction (mismatch: layer)	interaction (task:layer)	interaction (mismatch: task:layer)
statistic	$F_{1,52981}$=555.0	$F_{1,52981}$=247.5	$F_{1,52981}$=186.3	$F_{1,52981}$=24953	$F_{1,52981}$=2333	$F_{1,52981}$=274.9	$F_{1,52981}$=15870
p-value	p< $2e^{-16}$ (****)	p< $2e^{-16}$ (****)	p< $2e^{-16}$ (****)	p< $2e^{-16}$ (****)	p< $2e^{-16}$ (****)	p< $2e^{-16}$ (****)	p< $2e^{-16}$ (****)

III.6 Cognitive model-based analyses (ANOVA towards brain regions)

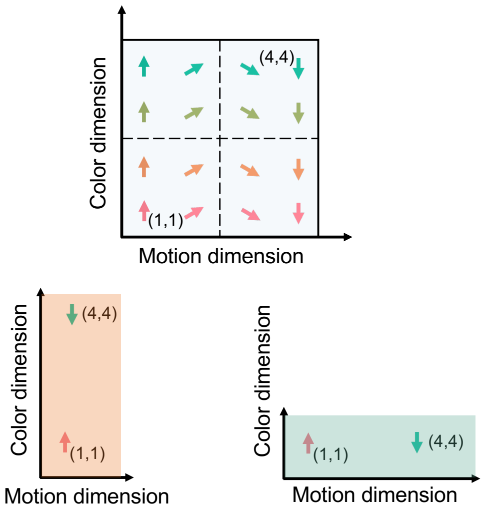

Figure S12: **Visual representations of attentional effect. The dissimilarities between stimuli pairs vary depending on the task context.** For instance, stimulus $(1, 1)$ and stimulus $(4, 4)$ have equal feature distance between motion and color dimension, however, in color-relevant context, see the left and orange shape, there would be dimensional stretching on color dimension between stimulus $(1, 1)$ and stimulus $(4, 4)$, that is, in the psychology space, the attention towards color-relevant information is greater than that towards motion-relevant information, and vice versa in motion-relevant context.

Table S14: A one-way repeated ANOVA was conducted to evaluate Spearman correlation score with a fixed factor of model (cognitive or baseline model) and a random effect for monkey subjects. The correlation scores are obtained from 5-fold cross-validation, while the attention weights are trained using 4 folds. For the cognitive model, these 4 folds are from the same task context, whereas for the baseline model, 2 folds are from the color task and 2 folds are from the motion task. A stronger correlation score was observed and found significant across all brain regions.

250 ms period data (ISI-distance)	PFC	FEF	LIP	IT	MT	V4	All
cognitive model score	0.18±0.09	0.22±0.11	0.17±0.11	0.11±0.10	0.19±0.14	0.15±0.11	0.26±0.09
baseline model score	0.11±0.10	0.13±0.12	0.10±0.10	0.01±0.09	0.11±0.12	0.03±0.15	0.18±0.10
statistic	$F_{1,37}$=4.835	$F_{1,37}$=6.311	$F_{1,37}$=4.725	$F_{1,37}$=11.75	$F_{1,37}$=4.312	$F_{1,37}$=10.23	$F_{1,37}$=5.96
p-value	0.0342 (*)	0.0165 (*)	0.0362 (*)	0.0015 (**)	0.0449 (*)	0.0028 (**)	0.0195 (*)

III.7 Cognitive model-based analyses (ANOVA towards LSTM layers)

Table S15: A three-way repeated ANOVA was conducted to evaluate the Spearman correlation score, with fixed factors of model (cognitive or baseline), layer number (1 to 6 LSTM layers), and training step (500-5000; in 500-step intervals, i.e., 1-10) and a random effect for simulations (4 simulations). The model type is treated as categorical variables, while the training step and the layer number are treated as continuous variables. The correlation scores were based on 5-fold cross-validation, while the attention weights are trained using 4 folds, similar to the procedure in brain analyses. For the cognitive model, 4 folds came from the same task, while for the baseline model, 2 folds came from the color task and 2 from the motion task. The results showed a significantly stronger correlation score, influenced by the model, training step, layer number, and their interactions (e.g., model and training step, model and layer number).

Three-way ANOVA	model (cognitive or baseline)	training step	layer number	interaction (model:step)	interaction (model:layer)	interaction (step:layer)	interaction (model:step:layer)
LSTMs	$F_{1,4789}$=6.2e4 p< $2e^{-16}$ (****)	$F_{1,4789}$=706.5 p< $2e^{-16}$ (****)	$F_{1,4789}$=4960 p< $2e^{-16}$ (****)	$F_{1,4789}$=583.6 p< $2e^{-16}$ (****)	$F_{1,4789}$=113.1 p< $2e^{-16}$ (****)	$F_{1,4789}$=93.05 p< $2e^{-16}$ (****)	$F_{1,4789}$=136.1 p< $2e^{-16}$ (****)

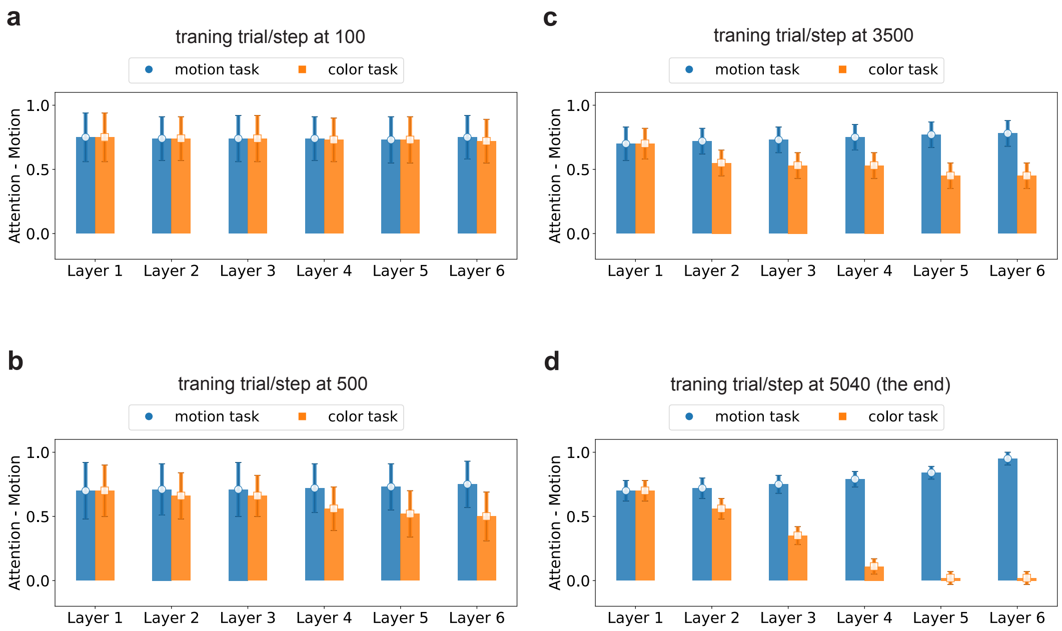

Figure S13: **The increasing attention weight through the training/learning of the CNN-LSTM model.** Similar to the procedure in brain analyses, we employed 5 folds, fitting the attention weight with the cognitive model in 4 folds from the same task. We showed the attention allocation towards the motion dimension during the training process at different training trials: 100 (**a**), 500 (**b**), 3500 (**c**), and the end of training (**d**).

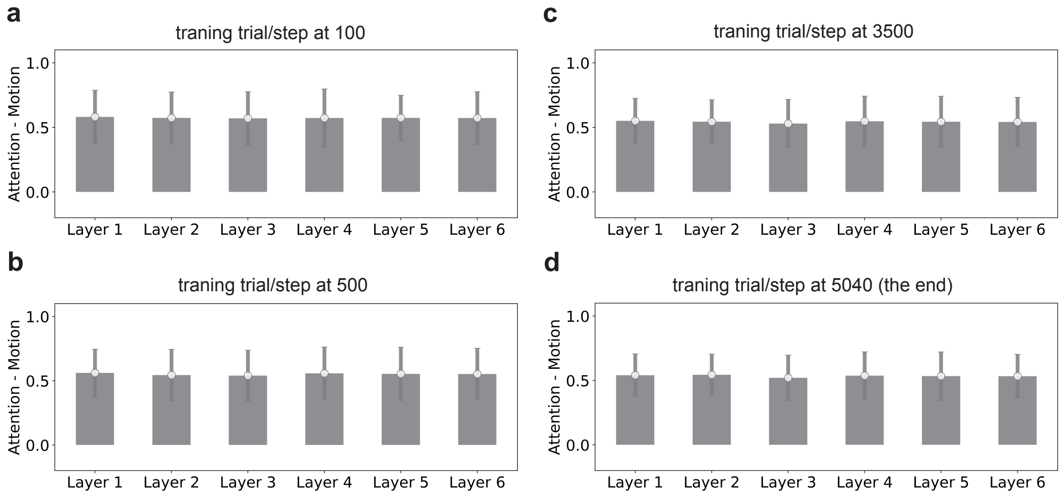

Figure S14: **The attention weight allocation using the baseline model was analyzed during the training process.** Similar to the procedure in brain analyses, we employed 5 folds, fitting the attention weight with the baseline model in 4 folds (2 folds from the color task and 2 folds from the motion task). The attention allocation towards the motion dimension was shown at different training steps: 100 (**a**), 500 (**b**), 3500 (**c**), and the end of training (**d**).

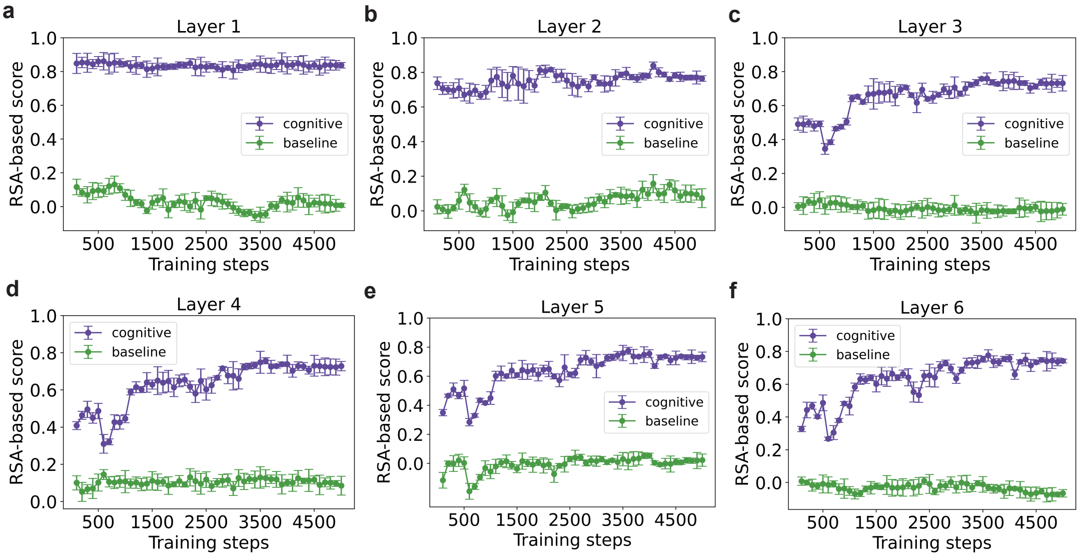

Figure S15: **The RSA-based correlation score during the training process.** We employed a 5-fold cross-validation approach to obtain correlation scores using attention weights fitted in both cognitive and baseline models. For the cognitive model, training utilized 4 folds from the same task, while the baseline model training incorporated 4 folds, equally split between the color task (2 folds) and the motion task (2 folds). We analyzed and presented the correlation scores for each LSTM layer throughout the training process. Panels **a–f** correspond to layers 1 through 6 of the model, respectively.

III.8 ANOVA analysis to compare the brain and the CNN-LSTM model

Table S16: To evaluate the RSA-based correlation score between brain and LSTM model layer across task contexts, we utilized a three-way repeated ANOVA with fixed factors of region (MT or V4, a categorical variable), task (motion or color relevant, a categorical variable), and layer number (1-6, a continuous variable) and a random effect for simulations (4 simulations). The interaction term of region and task is significant.

250 ms period data (ISI-distance)	region (MT or V4)	task (motion or color)	layer number	interaction (region:task)	interaction (region:layer)	interaction (task:layer)	interaction (region:task:layer)
F statistic	$F_{1,85}$=32.85 p=1.5e^{-7} (****)	$F_{1,85}$=91.34 p=4.0e^{-15} (****)	$F_{1,85}$=53.46 p=1.4e^{-10} (****)	$F_{1,85}$=670.5 p< 2e^{-16} (****)	$F_{1,85}$=6.49 p=0.0127 (*)	$F_{1,85}$=46.75 p=1.2e^{-9} (****)	$F_{1,85}$=28.73 p=7.0e^{-7} (****)

III.9 Additional results

Table S17: Permutation test comparing empirical and shuffled spike time distributions to validate the RSA analysis of spike timing. RSA-based correlation scores from empirical data were compared against permutation-based distributions (1000 independent runs of shuffling spike timing per trial) using two-sided Kolmogorov-Smirnov (KS) tests with false discovery rate (FDR) correction. All comparisons confirmed that the false discovery rate remained below 5%, indicating significant differences between empirical spike timing and chance-level distributions across both timing measures (ISI and SPIKE) and task contexts (motion and color).

**Condition**	**KS statistic**	**FDR corrected p value**
ISI – Motion	D=0.89	2.71×10^{-42}
SPIKE – Motion	D=0.92	3.56×10^{-52}
ISI – Color	D=0.68	8.63×10^{-38}
SPIKE – Color	D=0.69	1.02×10^{-42}

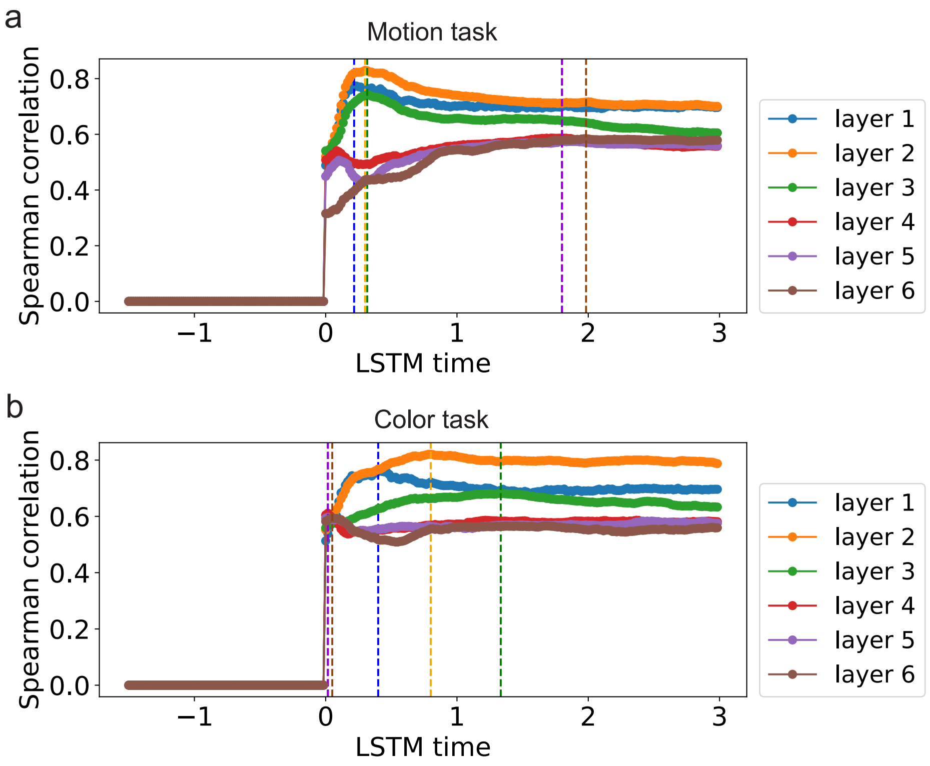

Figure S16: **Spearman correlation for CNN-LSTM model (representations measured by Euclidean distance) in the whole trial.** We compared the Spearman rank correlation between dissimilarity matrices (i.e., RDMs) constructed from the model representations (Euclidean distance) input from the 16 unambiguous stimulus items with an RDM derived from the experimenter-intended stimulus coordinates. Higher correlation indicates better agreement, shown for the motion task (**a**) and the color task (**b**). Spearman correlation is averaged over 1680 test trials. The colored dashed line indicates the point of highest correlation observed throughout the entire trial. Earlier model layers (e.g., 1, 2, and 3) reach their highest correlation earlier in the motion context, while later model layers (e.g., 4, 5, and 6) achieve their highest correlation earlier in the color context.

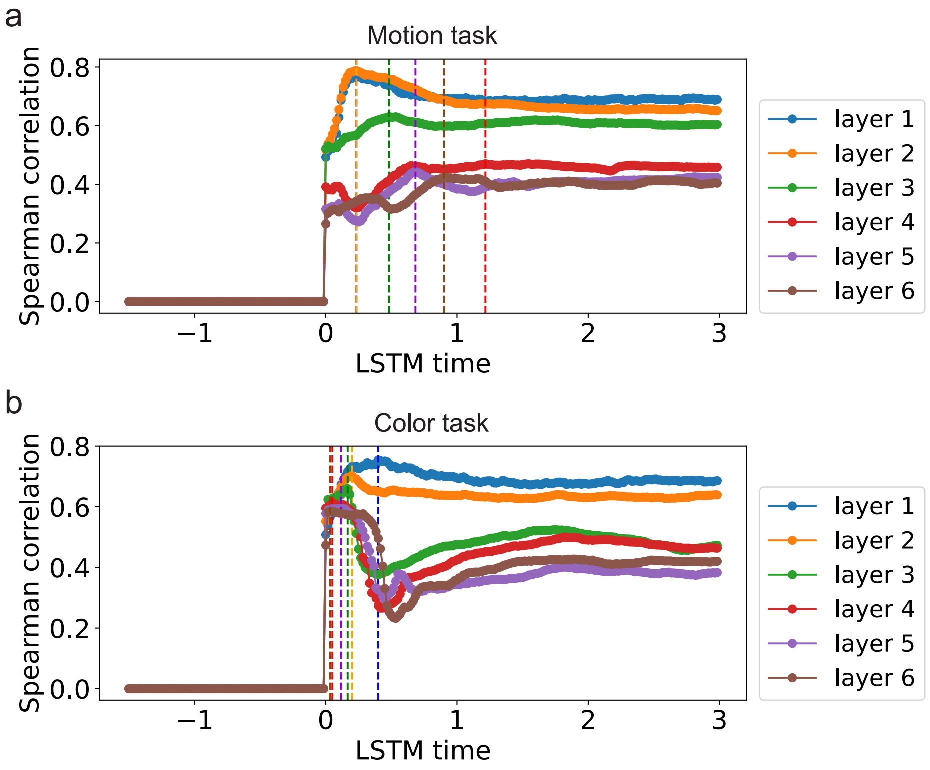

Figure S17: **Spearman correlation for CNN-LSTM model (representations measured by Cosine distance) in the whole trial.** We compared the Spearman rank correlation between dissimilarity matrices (i.e., RDMs) constructed from the model representations (Cosine distance) input from the 16 unambiguous stimulus items with an RDM derived from the experimenter-intended stimulus coordinates. Higher correlation indicates better agreement, with panel (**a**) corresponding to the motion task and panel (**b**) corresponding to the color task. Spearman correlation is averaged over 1680 test trials. The colored dashed line indicates the point of highest correlation during the entire trial. Earlier model layers (e.g., layers 1, 2, and 3) achieve the highest correlation sooner in the motion context, whereas later model layers (e.g., layers 4, 5, and 6) achieve the highest correlation sooner in the color context. This pattern is consistent as Supplementary Figure S16.

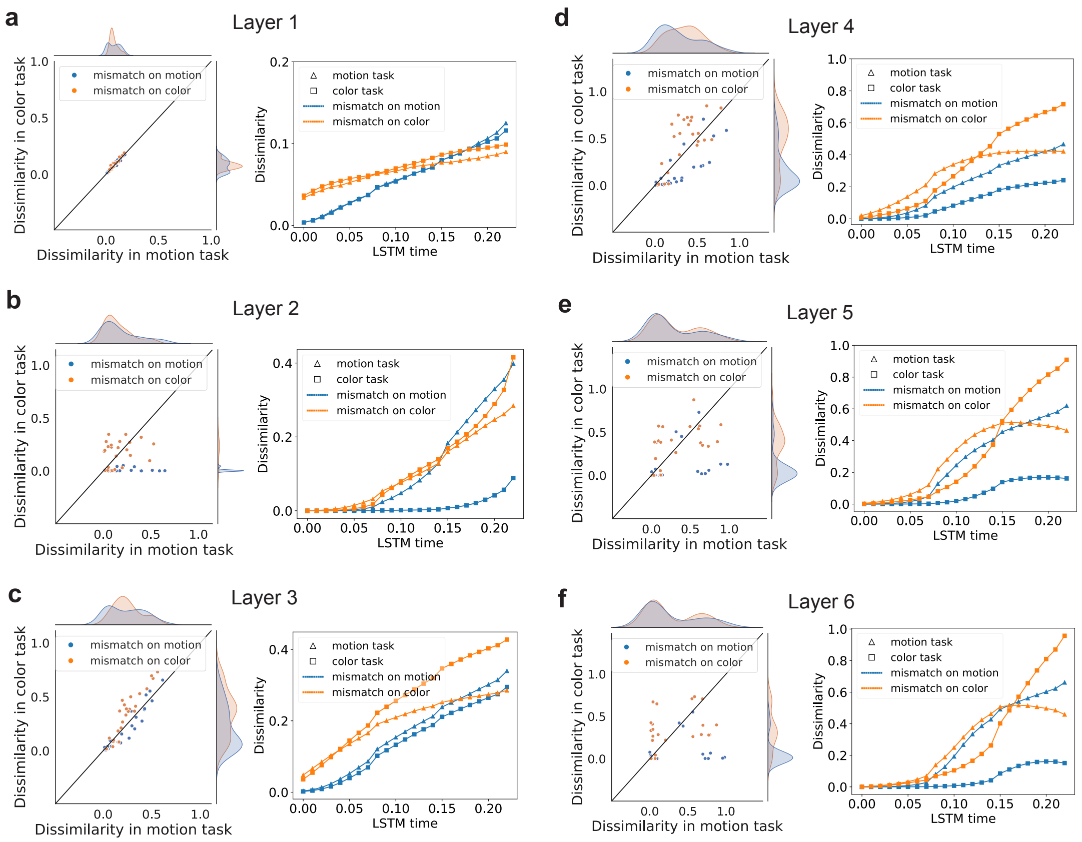

Figure S18: **Mismatching pairs in model representations measured by Cosine distance with a 50 ms sliding window.** Dissimilarity between item pairs mismatching on motion or color is increased when that dimension is task relevant. Shown are the results for LSTM layers 1–6 (**a**–**f**). Stimuli pairs that mismatch on task-relevant dimension become more different in task-relevant context than task-irrelevant context. The density distributions of these dissimilarities also indicate this task-modulation.

References

[1] Siegel, M., Buschman, T. J. & Miller, E. K. Cortical information flow during flexible sensorimotor decisions. *Science* **348**, 1352–1355 (2015).

[2] Kreuz, T., Haas, J. S., Morelli, A., Abarbanel, H. D. & Politi, A. Measuring spike train synchrony. *Journal of neuroscience methods* **165**, 151–161 (2007).

[3] Kreuz, T., Chicharro, D., Houghton, C., Andrzejak, R. G. & Mormann, F. Monitoring spike train synchrony. *Journal of neurophysiology* **109**, 1457–1472 (2013).
